# A theoretical examination of the relative importance of evolution management and drug development for managing resistance

**DOI:** 10.1098/rspb.2014.1861

**Published:** 2014-12-22

**Authors:** Nathan S. McClure, Troy Day

**Affiliations:** 1 Department of Biology, Queen's University, Kingston, Ontario, Canada K7L 3N6; 2 Department of Mathematics and Statistics, Queen's University, Kingston, Ontario, Canada K7L 3N6

**Keywords:** drug resistance, pharmaceuticals, chemotherapy, treatment strategies

## Abstract

Drug resistance is a serious public health problem that threatens to thwart our ability to treat many infectious diseases. Repeatedly, the introduction of new drugs has been followed by the evolution of resistance. In principle, there are two complementary ways to address this problem: (i) enhancing drug development and (ii) slowing the evolution of drug resistance through evolutionary management. Although these two strategies are not mutually exclusive, it is nevertheless worthwhile considering whether one might be inherently more effective than the other. We present a simple mathematical model that explores how interventions aimed at these two approaches affect the availability of effective drugs. Our results identify an interesting feature of evolution management that, all else equal, tends to make it more effective than enhancing drug development. Thus, although enhancing drug development will necessarily be a central part of addressing the problem of resistance, our results lend support to the idea that evolution management is probably a very significant component of the solution as well.

## 1. Introduction

In our efforts to manage infectious diseases, the usefulness of newly developed drugs has been undermined by the evolution of drug resistance [1–6]. This pattern has been repeated so often in the history of some diseases that the evolution of resistance is now considered inevitable [6–14].

The evolution of resistance to any particular drug is not problematic provided that an alternative drug is available. What matters is the rate at which drug resistance evolves relative to the rate at which new drugs are brought into use. Consequently, there are two complementary approaches to ensuring the availability of effective drugs: (i) increasing the rate of drug arrival through enhanced drug development and (ii) increasing the time it takes for drug resistance to evolve through better evolution management.

Approaches for increasing the rate of drug development are probably familiar, and include developing screening technology for new compounds, developing new classes of antimicrobial agents, improving patent protection of new pharmaceuticals, speeding up the drug approval process and expanding public–private partnerships for drug development [15–20]. Approaches for slowing the evolution of resistance are perhaps less familiar, but include developing new diagnostic technologies and strategies for reducing the inappropriate use of antimicrobials, formulating strategies to reduce transmission and spread of infection to avoid using antimicrobials, determining when and where existing drugs should be used in combination versus as sequential monotherapies, as well as determining the optimal dose and timing of deployment for these existing drugs [1,2,5,6,21–26].

These two facets of the problem (i.e. enhancing drug development and slowing evolution) are not mutually exclusive, and any comprehensive strategy for dealing with drug resistance will incorporate both. In some situations, enhancing the rate of drug development might be easiest while in others it might be easier to focus on the evolutionary side of the problem (for example, if it is too difficult to provide adequate incentivization for drug development). Even so, however, it is worthwhile asking whether one approach is somehow inherently more effective than the other. For example, in the simplest situation, where it is equally possible to enhance drug development as it is to slow evolution, does one of these interventions nevertheless have a greater effect?

To answer this question, we need to determine the benefits, in terms of ensuring effective drug availability, of increasing the rate of drug arrival through enhanced drug development and of slowing the rate of evolution through better resistance management. At one level, the answer to this question is obvious. If there is an upper limit to the number of drugs that can be developed for a particular disease, then at some point drug development will become effectively impossible. This would leave slowing evolution as the only option. But what if we are not yet facing this limitation on drug development? Here, we explore this question through the development of simple mathematical models.

## 2. Material and methods

### (a) Model

**Figure 1.**
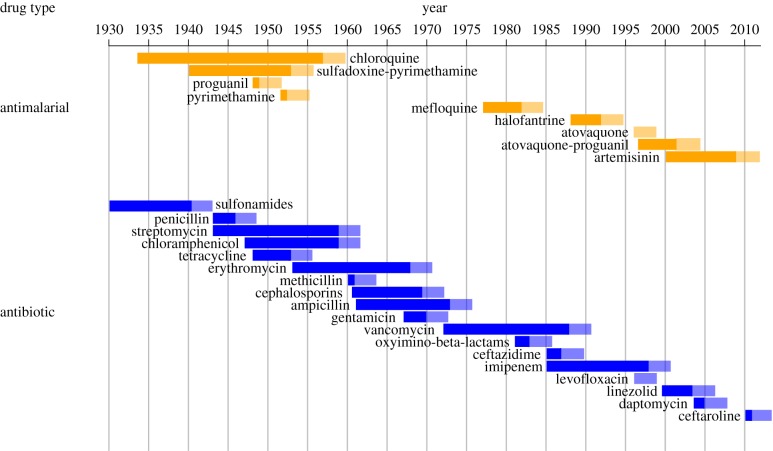
Timeline for antimalarials and antibiotics [6–14]. The times of drug introduction and the subsequent evolution of resistance are indicated by the ends of the bars. The faded region is meant to show that the first observation of resistance does not absolutely equate with complete loss of treatment efficacy. (Online version in colour.)

Historical data for the time of introduction of some antimalarial and antibiotic drugs, as well as the evolution of resistance to these, are presented in figure 1. The processes underlying these data are complex, with both geographical and temporal variation in the drugs and the diseases that they are used to treat. Consequently, not too much should be read into the precise dates of any specific drug (see the electronic supplementary material). That said, the data do provide a qualitative illustration of what has been called the ‘drug resistance treadmill’ [27]. New drugs are continually brought into use, followed by the evolution of resistance after some variable period of time.

Our goal is to use the broad qualitative features of figure 1 as a guide for constructing some simple mathematical arguments. We do not attempt to develop a model specific to any particular drug or disease but instead focus on modelling some broad features that are likely to underlie the drug resistance treadmill in general. For illustrative purposes, however, we will highlight some of the general results with specific parameter values taken from data on antimalarial drugs [8–14].

To begin, we define a ‘drug’ as any whole drug therapy, which might consist of one or multiple active ingredients. The ‘drug portfolio’ is defined as the collection of existing drugs that are effective against the specific type of infection of interest. For simplicity, we assume that a single drug is used at any given time, and its use is continued until resistance to this drug has appeared and reached some threshold frequency in the population (e.g. 10% treatment failure rate for antimalarials [21]). At this point, the drug's ability to provide effective treatment is compromised and so its use is effectively discontinued. In order to continue to provide effective treatment for the disease, another drug from the drug portfolio, if available, is then brought into use (similar to the ‘wait-and-switch’ strategy for malaria [25]). During this process, new drugs are assumed to be in various stages of development and are occasionally brought into the drug portfolio.

The above characterization of the drug resistance treadmill might seem like an oversimplification of reality. After all, in many cases, multiple drugs are in use simultaneously, and drugs are not always entirely abandoned when resistance to them is common. Instead, there can be a complex global geographical mosaic of levels of resistance and patterns of drug use over time [28]. Despite this complexity, however, we develop the above caricature of the resistance treadmill for two reasons. First, it captures two of the main processes at play in any drug resistance treadmill: the development of new drugs and the loss of these drugs through resistance. Second, even this very simple setting produces some surprising results that are not immediately obvious. Furthermore, we will argue that the insights provided by this very simple model are likely to hold more generally (also see the electronic supplementary material for details of results that relax these assumptions).

**Figure 2.**
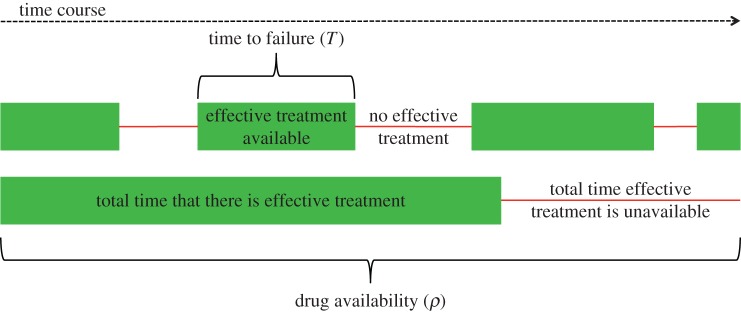
Schematic of the model. A timeline defining the time to failure, *T*, and drug availability, *ρ*. ‘Effective treatment available’ means that there is at least one effective drug in the portfolio. (Online version in colour.)

Figure 2 provides a schematic of the overall drug resistance treadmill. Drug arrival into the drug portfolio and the evolution of resistance both occur stochastically, resulting in periods of time for which effective treatment is available (i.e. when there is at least one effective drug in the drug portfolio). We refer to the length of time during which effective treatment is available as the ‘time to failure’, *T*. These periods are separated by time intervals during which treatment is compromised by drug resistance (i.e. when all current drugs are affected by drug resistance; figure 2). For example, the appearance of pan-resistant *Klebsiella pneumoniae* and multidrug-resistant *Mycobacterium tuberculosis* suggests that we might well have exceeded the time to failure for these diseases, and may now be heading into a period in which no completely effective treatment is available [29–31].

The expected time to failure can be viewed as the product of the expected number of drugs used before failure occurs and the expected lifespan of each of these drugs. This decomposition will be important for understanding the results, and we will return to it in §4 (see equation (4.1)). We will also be interested in the long-run fraction of time that effective drugs are available, and we refer to this as the ‘drug availability’ (figure 2).

The model is completely specified by the time between drug arrivals and the time to evolve resistance. Analysis of a large dataset for pharmaceutical production from companies during the period 1950–2008 has shown that the annual output of new drugs is approximately Poisson distributed with a constant rate parameter [32]. This implies that the time between new drug arrivals is exponentially distributed. We note however that these data include all ‘new molecular entities' and so are not restricted to drugs specific to the treatment of infectious diseases. Likewise, a ‘new molecular entity’ does not correspond exactly to our definition of a drug given above. Nevertheless, an exponential distribution is also consistent with the limited data available in figure 1 (see the electronic supplementary material), and therefore we start our analysis by making this assumption. We define the random variable *D* to be the time between drug arrivals into the drug portfolio, and assume that *D* is exponentially distributed with rate parameter, *α*. The expected time between drug arrivals is therefore *E*[*D*] = 1/*α* (e.g. 8.3 years for antimalarials; see the electronic supplementary material).

We define *L* as the time to evolve resistance (i.e. the drug lifespan), and it represents the time between when a drug is first used and when resistance to the drug reaches a threshold frequency. We assume that this lifespan is determined by the evolution of resistance, and therefore different evolution management strategies will result in different drug lifespans. Little is currently known about the distribution of *L*, but we can make some progress by examining the data from figure 1. Analyses suggest that, for some diseases at least (e.g. malaria), *L* is also approximately exponentially distributed (see the electronic supplementary material). There are many caveats associated with this conclusion, however, and therefore we consider two scenarios. First, we suppose that *L* is exponentially distributed with rate parameter *β*. The expected drug lifespan is therefore *E*[*L*] = 1/*β* (e.g. 5 years for antimalarials; see the electronic supplementary material). Second, we consider a case where the distribution of *L* is left arbitrary.

### (b) Slowing resistance evolution versus enhancing drug development

We begin the analysis by assuming that the average time between drug arrivals is larger than the average drug lifespan (i.e. *E*[*D*] > *E*[*L*]). This assumption is relaxed when we consider a variable rate of drug arrival (see §2*c*). This implies that, with probability 1, there will be periods of time when effective drugs are available as well as periods of time when they are not (figure 2).

Two interventions are explored: (i) decreasing the expected time between drug arrivals *E*[*D*] through enhanced drug development and (ii) increasing the expected drug lifespan *E*[*L*] through better evolution management. For completeness, we explore both additive and multiplicative changes in each. In the additive case, we consider decreasing *E*[*D*] by an additive amount or increasing *E*[*L*] by the same amount. In the multiplicative case, we consider decreasing *E*[*D*] by a factor or increasing *E*[*L*] by the same factor. We focus on two main performance measures: the time to failure, *T*, which is a random variable, and the drug availability (i.e. the long-run proportion of time an effective drug is available), *ρ* (figure 2).

### (c) Numerical simulations

Although we can make analytical progress using the above assumptions, it is important to assess the robustness of our conclusions to changes in these assumptions. To this end, we perform numerical simulations that explore two other possibilities. The first explores the consequences of having a distribution of drug inter-arrival times, *D*, that is not exponential. To do so, we instead use a gamma distribution for *D*. To perform numerical calculations, we also then need to specify the distribution of drug lifespans *L*, and we use a gamma distribution for this as well.

The second numerical simulation relaxes the assumption that the rate of drug arrival is constant and instead supposes that this rate is inversely related to the drug portfolio size. This allows us to explore the plausible situation in which the rate of drug development responds to demand. To accomplish this, we used two rates of drug arrival: a ‘normal rate’, denoted by *α*, and a ‘fast rate’, denoted by *θ*. At any one time only one rate of drug arrival occurs, but the one that is in effect depends on the number of drugs remaining in the drug portfolio at the time of the last drug arrival. The time between drug arrivals is still assumed to be exponentially distributed, but the rate parameter now changes between a ‘normal rate’ (*θ*) of drug arrival. In this simulation, we also used exponentially distributed drug lifespans, *L*, with rate parameter *β*.

## 3. Results

### (a) Analytical results

**Figure 3.**
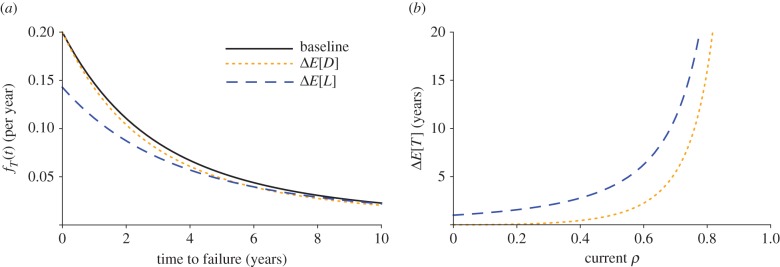
Effects on time to failure from enhancing drug development and slowing evolution with estimates from antimalarial data (see the electronic supplementary material). (*a*) The effect on the time to failure density *f_T_*(*t*) when the average drug inter-arrival time is reduced by 2 years (Δ*E*[*D*]) and the average drug lifespan is extended by 2 years (Δ*E*[*L*]) compared with baseline conditions (*E*[*L*] = 5 years, *E*[*D*] = 8.3 years). (*b*) The change in expected time to failure (*E*[*T*]) resulting from additive perturbations in *E*[*L*] and *E*[*D*] plotted for varying current drug availability (*ρ*). (Online version in colour.)

When the distribution of the time to evolution, *L*, and the drug inter-arrival time, *D*, are both exponential, an explicit equation can be derived for the probability density of the time to failure *T*. We have3.1
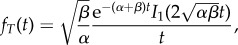
where *I*_1_(*x*) is a modified Bessel function of the first kind (see the electronic supplementary material). Likewise, drug availability is given by3.2
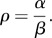
We can see that decreasing the mean time between drug arrivals (i.e. decreasing *E*[*D*] = 1/*α*) or increasing the mean time to evolve resistance (i.e. increasing *E*[*L*] = 1/*β*) both shift probability mass in equation (3.1) from low to high values of *T.* However, changes in the mean time to evolve resistance do so to a greater extent (figure 3*a*). This is true regardless of whether the changes are additive or multiplicative (see the electronic supplementary material). Likewise, drug availability, *ρ*, is also increased more through an additive change in the mean time to evolve resistance, whereas both interventions have identical effects on *ρ* when the changes are multiplicative (see the electronic supplementary material).

When the distribution of time to evolve resistance is left arbitrary, we can still obtain an equation for an integral transform of the distribution of time to failure, from which we can calculate any of its moments (see the electronic supplementary material). We focus here on the first moment (i.e. expected time to failure), although we analyse the second moment in the electronic supplementary material. The first moment is3.3
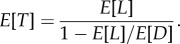
The expression for drug availability in this case is identical to equation (3.2) (see the electronic supplementary material). Again we can see that the expected time to failure, *E*[*T*], increases more with a change in *E*[*L*] than it does with a change in *E*[*D*] (figure 3*b*), and again this is true regardless of whether the changes are additive or multiplicative. The conclusions about drug availability are identical to the case where the distribution of time to evolution is exponential.

### (b) Numerical simulations

**Figure 4.**
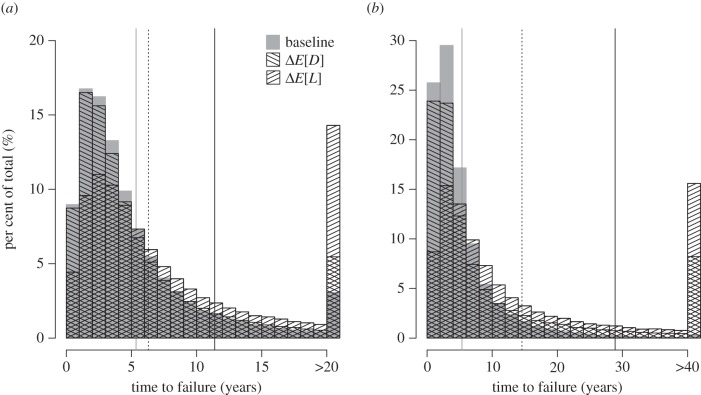
Histogram of time to failure from numerical simulations of a gamma-distributed drug inter-arrival time, *D*, and drug lifespan, *L*. Baseline conditions are given as *k* = 2, *α* = 1/5, *β* = 1/2. (*a*) The effect on time to failure from an additive change decreasing *E*[*D*] by 2 years (*k* = 2, *α* = 1/4; Δ*E*[*D*]) or increasing *E*[*L*] by 2 years (*k* = 2, *β* = 1/3; Δ*E*[*L*]). (*b*) The effect on time to failure from a multiplicative change halving *E*[*D*] (*k* = 2, *α* = 2/5; Δ*E*[*D*]) or doubling *E*[*L*] (*k* = 2, *β* = 1/4; Δ*E*[*L*]). The last bar of the histogram represents all remaining values of time to failure in the simulation. Vertical lines represent average length of time to failure for baseline conditions (grey solid line) and perturbations decreasing *E*[*D*] (black dotted line) or increasing *E*[*L*] (black solid line).

In the first numerical exploration of the robustness of our results, we used a gamma-distributed drug inter-arrival time, *D*, and gamma-distributed drug lifespan, *L*. The gamma distribution is parametrized by a shape parameter, *k*, and a rate parameter, which we denote by *α* and *β* for the drug inter-arrival time and drug lifespan, respectively. The expected time between drug arrivals is therefore *E*[*D*] = *kα*. Similarly, the expected time to evolve resistance is *E*[*L*] = *kβ*. We numerically calculated time to failure and drug availability for an additive change and a multiplicative change that increased *E*[*L*] or that decreased *E*[*D*], and compared the results. Figure 4 shows how the distribution of time to failure changes as a result of these two manipulations. In both cases, time to failure increases; however, we observe a greater increase in the average time to failure when *E*[*L*] is increased than when *E*[*D*] is decreased. For an additive change, there was also a greater increase in drug availability when *E*[*L*] increased than when *E*[*D*] decreased, while for a multiplicative change, drug availability was increased by the same amount. This agrees with the analytical results (see the electronic supplementary material).

**Figure 5.**
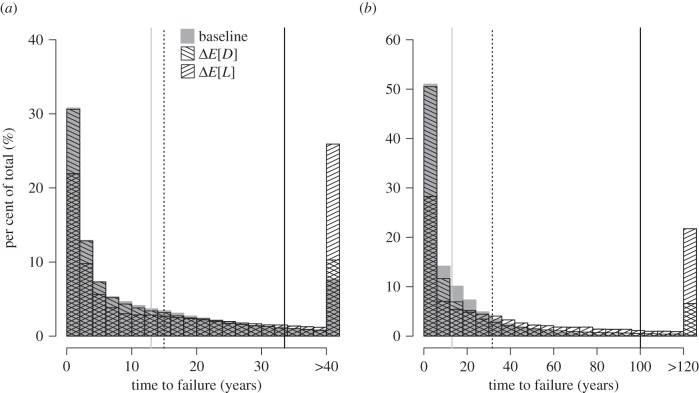
Histogram of time to failure from numerical simulations of a variable rate of drug development as inversely related to the size of the drug portfolio. Baseline conditions are given as *α* = 1/10, *β* = 1/4. (*a*) The effect on time to failure from an additive change decreasing *E*[*D*] by 2 years (*α* = 1/8; Δ*E*[*D*]) or increasing *E*[*L*] by 2 years (*β* = 1/6; Δ*E*[*L*]). (*b*) The effect on time to failure from a multiplicative change halving *E*[*D*] (*α* = 1/5; Δ*E*[*D*]) or doubling *E*[*L*] (*β* = 1/8; Δ*E*[*L*]). The same ‘fast rate’ of drug development (*θ* = 1/3) was used in the simulations when there were two or fewer drugs remaining in the drug portfolio at the time of the last drug arrival. The last bar of the histogram represents all remaining values of time to failure in the simulation. Vertical lines represent average length of time to failure for baseline conditions (grey solid line) and perturbations decreasing *E*[*D*] (black dotted line) or increasing *E*[*L*] (black solid line).

In the second numerical exploration, we relaxed the assumption of a constant rate of drug arrival and examined the situation in which drug arrival is inversely related to drug portfolio size. Time to failure and drug availability were numerically calculated for an additive change that increased *E*[*L*] or decreased *E*[*D*] under the ‘normal rate’ of drug arrival. The same ‘fast rate’ of drug arrival was used throughout the calculations when there were two or fewer drugs remaining in the drug portfolio. Input parameter values were chosen such that the average time between drug introductions under a ‘fast rate’ of drug arrival was less than the average drug lifespan, relaxing the earlier assumption that *E*[*D*] > *E*[*L*]. Figure 5 shows how time to failure increased as a result of changing *E*[*L*] or *E*[*D*]. There was a substantially larger increase in average time to failure when *E*[*L*] was increased, as was found in the simpler analytical results. The increase in *E*[*L*] also yielded a greater increase in drug availability than a corresponding decrease in *E*[*D*] (see the electronic supplementary material).

## 4. Discussion

The chemotherapeutic treatment of infectious diseases invariably leads to the evolution of drug resistance. This problem has been recognized for a long time but, as with many things, we as a society have tended to confront the issue only when forced to do so. The situation now appears to be reaching this point. We are facing the prospect of a lack of treatment availability for some infections, and there is growing concern about the slow rate at which new antimicrobials are entering the drug portfolio [33,34]. The development of new pharmaceuticals is thus becoming increasingly urgent.

It would, of course, be prudent to attempt to deal with the problem of resistance even in times when effective treatment is readily available. Whether or not we eventually end up facing the failure of all available treatment for a disease depends upon the balance between the rate of drug arrival and resistance evolution during these ‘times of plenty’. Thus, optimizing the drug supply system as a whole over the long term should be a primary goal. The results presented here are an attempt to make some modest progress in this direction.

The full scope of the drug resistance problem is complex and involves scientific, economic, political and sociological dimensions [1,2,5,28,35]. No single strategy is likely to be successful in addressing these issues; nevertheless, it is often useful to examine different facets of the problem in isolation. Once these isolated components are more fully understood, we can begin to piece them together and to develop a deeper understanding of the entire issue.

In this article, we have examined one part of the problem by asking how the relative impacts of investing in drug development and investing in better evolution management compare in terms of their effects on maintaining a supply of effective treatment. Our results suggest that, all else equal, increasing the lifespan of drugs through better resistance management has a greater effect than does a corresponding decrease in the time it takes to develop new drugs.

Our analysis was intentionally based on a greatly simplified model of the drug resistance treadmill that is meant to capture only the core qualitative features of drug development and resistance evolution. The goal of this simplification was to lay bare any inherent differences in how the introduction of new drugs through development and their ‘removal’ through resistance evolution affect the long-term availability of effective treatment. By doing so, we can more easily understand the mechanisms underlying these differences and thus better judge whether the differences are likely to be relevant in more realistic settings.

What drives the inherent advantage of evolution management in our model? Both types of interventions (i.e. enhancing evolution management and enhancing drug development) increase the chance that new drugs will be developed before the system as a whole fails and there is no effective treatment available. Increasing the time to evolve resistance does so because it extends the window of opportunity for a new drug to be developed before failure occurs. Decreasing the time between drug arrivals does so because, on average, more drugs will arrive in a given period of time. In fact, when the changes for each intervention are multiplicative, it can be shown that, on average, the same number of drugs will arrive and be used before failure occurs in both cases. The difference between them lies in how the interventions affect the lifespan of each drug. Recall that we can express the mean time to failure, *E*[*T*], as the product of the expected number of drugs that are used before failure occurs and the expected lifespan of each drug. Mathematically,4.1
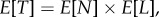
where *N* is the number of drugs used before system failure. As already described, multiplicative changes in both interventions have identical effects on *E*[*N*]. But changes in the time to evolve resistance also affect *E*[*L*], whereas changes in drug arrivals do not. As a result, increasing the expected time to evolve resistance has a compounding effect that is absent when changing the drug inter-arrival time. Moreover, this effect is even greater when we consider additive changes in each intervention because changing the time to evolve resistance then increases *E*[*N*] more than does changing the time between drug arrivals.

What about drug availability? Drug availability is the long-run proportion of time for which there is at least one effective drug available. Hence, even though there is a greater increase in time to failure when evolution is slowed, it is important to consider how enhancing the rate of drug arrival reduces the fraction of time for which there is no effective drug. If the changes are multiplicative, the increase in drug availability that arises from slowing the evolution of resistance is equivalent to the increase in drug availability that comes from speeding up drug arrivals. However, if the change is additive, then drug availability increases more when resistance evolution is slowed.

Analysis of the numerical simulations gave the same result that slowing the rate of evolution has a greater effect on time to failure and drug availability than increasing the rate of drug arrival. As one possible generalization of the assumption of exponentially distributed *D* and *L*, the first case used gamma-distributed times between drug arrivals and drug lifespans. In the second case, the rate of drug arrival was varied to study the effect of having drug development respond to demand, as well as allowing for the possibility for the mean drug lifespan to exceed the mean time between drug arrivals (i.e. *E*[*D*] < *E*[*L*]). These results show that, at least under the studied conditions, relaxing some of our initial model assumptions does not alter the value of increasing drug lifespan on ensuring the availability of effective treatment.

Our model assumes that, when available, drugs are used one at a time. In reality, multiple drugs are often used simultaneously at the population level, although perhaps in different geographical regions [36,37]. How would this affect our conclusions? Modelling the simultaneous use of multiple drugs is difficult because one needs to specify how the rate of resistance evolution to each drug is affected by all of the specific drugs that are in use, and virtually no data are available on this.

Interestingly, however, there are some situations in which simultaneous multiple drug use results in a model that is formally identical to the model presented here. In such situations, the results obtained in our simplified model then apply directly to this more complex setting. This occurs, however, only when there is a sort of ‘conservation of evolution’ (see the electronic supplementary material), and this type of conservation is unlikely to occur in all situations. Nevertheless, it does serve as a useful baseline for understanding how the results might change under different assumptions.

We also wish to stress that, although the main model presented is highly simplified, the phenomenon that it reveals and that is highlighted in equation (4.1) is likely to operate under much more general conditions. For example, if multiple drugs are in use simultaneously then, even though the process might be much more complicated, each drug will still spawn the evolution of resistance (possibly in way that interacts with the evolution of resistance to other drugs). Regardless of the details, however, both better evolution management and faster drug arrivals will still increase the number of drugs used before the system ultimately fails (the factor *E*[*N*] in equation (4.1)). But, by definition, evolution management will also still increase the lifespan of each drug, thereby giving evolution management the same compounding benefit seen in our simple model (the factor *E*[*L*] in equation (4.1)). Of course, other factors could also come into play in these more complex situations, making it less clear whether the overall effect is still greatest for evolution management. For example, it is possible that the increase in *E*[*N*] that results from enhanced drug development is greater than that from better evolution management in some more realistic settings, and this is an interesting area for future research. But, even so, evolution management should still have an inherent advantage *all else equal* because of the compounding effect seen in equation (4.1).

How do the ideas presented here relate to specific cases of the so-called drug resistance treadmill? In the case of HIV, for example, the success of treatment is due to many factors, one of which is the expansion of its drug portfolio [3]. However, the discovery that combination therapy can greatly slow the evolution of drug resistance within individual patients has been instrumental in successfully ensuring effective treatment [3,38]. This can be viewed as a premier example of enhanced evolution management at the individual level. In fact, we might view the emergence of resistance and subsequent failure of treatment at the individual patient level as a drug resistance treadmill as well. As initial chemotherapeutic agents fail, new treatments are used until the portfolio of available drugs is exhausted. Although developing new monotherapies faster would certainly help the situation, our model suggests that the evolution management strategy of using combination therapy might well have had an inherent advantage over such an approach.

In a similar way, resistance profiling in HIV-1 treatment is another example of enhanced evolution management at the individual level, because it helps to guide appropriate drug choices that avoid favouring resistance [3,38–40]. Again, although our abstract model does not explicitly incorporate the specific details of this strategy, it does suggest that such an approach might have inherent advantages [3,38–41].

Treatment of *M. tuberculosis* also uses a combination of anti-tuberculars with evidence of resistance to individual drugs [3]. Available treatment is effective against susceptible strains; however, multidrug-resistant and extensively drug-resistant infection pose serious complications [3,15,41,42]. Current recommendations include production of combination therapies in place of monotherapies, improved understanding of anti-tubercular treatment in association with resistance and HIV-1 infection, and diagnostic testing for drug-resistant strains [3,41–44]. It is not possible to measure the exact value of changes in drug lifespan and drug development for treatment of *M. tuberculosis* using our simplified model. However, our model presumably again captures an important process that emphasizes the significance of evolution management in ensuring effective treatment [1,3,30,41–43,45].

In the case of malaria, drug resistance is perhaps the greatest challenge to global control and elimination [3]. The process we have identified by way of our simplified model suggests that slowing drug-resistance evolution is advantageous to ensuring the availability of effective antimalarials. Doing so might involve using multiple first-line therapies to extend the lifespan of each drug by slowing the spread of drug-resistant infection, optimizing drug combinations (as well as drug dose and duration) to weaken the selective advantage of drug-resistant parasites, developing diagnostic technologies to guide appropriate drug use, formulating guidelines and surveillance programmes to use drugs prudently in prophylaxis and treatment, and preventing transmission through effective vector control [21,24,46,47]. Indeed, many initiatives of this nature are currently under way, which may prove useful in lengthening the lifespan of available therapies as well as new antimalarials as they are developed.

Importantly, our analysis does not consider how investments for enhancing drug development and slowing evolution can be delivered to the system. Additive and multiplicative changes were used to provide a fair comparison of the importance of speeding up drug arrivals relative to slowing resistance evolution when all else is equal. However, it might be less costly or easier to change drug lifespan than to change drug development, or vice versa, and this is an interesting area for future research [4,17,18,24]. Drug development has associations with the private sector, and so increasing the rate at which drugs are brought to market involves incentivizing pharmaceutical companies. On the other hand, much of the research on slowing resistance evolution may depend on funding from public grant money that is held at universities and other research institutions, and therefore this work is subject to very different market forces. For these reasons, it is not immediately clear how easily drug development can be changed relative to evolution management.

Even though we provide evidence that slowing resistance evolution can be more beneficial to the effective management of antimicrobial resistance than speeding up drug development, we stress that this in no way negates the importance of pharmaceutical innovation and drug development. Indeed, as we mentioned in §1, the two approaches to resistance management are not mutually exclusive. In many ways, slowing the evolution of drug resistance is fundamentally linked to drug development. For example, investment and advancement in drug development might lead to smarter drugs with enhanced efficacy and greater longevity. Thus, our intention is not to suggest that there is necessarily an antagonism between the two approaches. The results do demonstrate, however, that a strong emphasis on evolution management might yield promising results for this pressing problem. A major challenge is therefore to provide incentives for new drug development and research of antimicrobial stewardship strategies for which resistance evolves less quickly.

## Supplementary Material

Supplementary Material for A theoretical examination of the relative importance of evolution management and drug development for managing resistance
