# Supplementary material for: A theoretical examination of the relative importance of evolution management and drug development for managing resistance
Source: Proc Biol Sci. 2014 Dec 22;281(1797):20141861. doi: 10.1098/rspb.2014.1861 (PMC4240990; doi:10.1098/rspb.2014.1861)
Supplement: Supplementary Material for A theoretical examination of the relative importance of evolution management and drug development for managing resistance [file rspb20141861supp1.pdf]

Supplementary Material

for

A theoretical examination of the relative importance of evolution management and drug development for managing resistance

Nathan S. McClure[1] and Troy Day[1,2]

1. Department of Biology, Queen's University, Kingston, ON, K7L 3N6, Canada
2. Department of Mathematics and Statistics, Queen's University, Kingston, ON, K7L 3N6, Canada

Contents

1 – Data **3**
Approach 1: Dividing overlapping periods by the number of drugs *3*
Approach 2: Time to evolve resistance from earliest drug arrival to first drug failure... *4*
Simulating the current and future state of antimalarial supply *4*

2 – Analysis **4**
Exponentially Distributed Time to Evolve Resistance *4*
Arbitrary Distribution for the Time to Evolve Resistance *6*
Second Moment of Time to Failure *8*

3 – Numerical Simulations **9**
Gamma Distributed Drug Inter-arrival Time and Drug Lifespan *9*
Variable Rate of Drug Arrival *9*

4 – Generalizing to Multiple Drugs **10**

5 – Supplementary Tables **12**

6 – Supplementary Figures **15**

1 – Data

Data were collected from various sources to create a timeline of drug supply for antimalarials and antibiotics (Table S1). The data consist of the date of drug introduction, and the date of first recorded resistance. When there existed multiple estimates for the same drug from a single source, we report the earliest date that the drug was introduced and the earliest date that resistance was first observed (this occurred when there was region-specific data on drug introduction or resistance). While it is reported that resistance has developed to every antimalarial (with the possible exception of artemisinin therapies [3]), we excluded data on Lapdap, Amodiaquine, and Primaquine because the determination of drug resistance for these antimalarials was inconclusive (e.g., due to a lack of evidence or because of confounding factors in treatment failure such as cross resistance, side-effects, compliance and drug withdrawal [49-51]).

We also used the available antimalarial data to give an example parameterization of the model. We stress, however, that this is intended merely as an illustrative case. All of the results reported in the main text are general, and independent of the disease in question as well as the specific parameter values used. We nevertheless chose to include an example parameterization based on data because it helps to clarify the relevance of the general results and to make them more concrete.

Coming up with a suitable parameterization from the data is difficult because our simplified model lacks some of the features of the real data. Specifically, in the data many drugs have overlapping lifespans (elapsed time from date of drug introduction to date of observation of resistance). This is because more than one drug is used at a time during these periods. Our simple model assumes that one drug is used at a time (although Section 4 generalizes these results) and therefore we need to extract suitable estimates from the data under this assumption. For example, we cannot use the observed drug lifespans since the sum of individual drug lifespans would be greater than the observed time to failure, misrepresenting the current state of drug supply. As a result we employed two different approaches (see below) to estimate the time to evolve resistance from the data. The distribution of time to evolve resistance is almost identical under both approaches, and resembles an exponential distribution with rate parameter $\beta = 0.2$ (see Figure S1). The distribution of time between drug arrivals also resembles an exponential distribution with rate parameter $\alpha = 0.12$, supporting an assumption based on results from [31] (see Figure S1).

Approach 1: Dividing overlapping periods by the number of drugs

We can estimate the time to resistance of individual drugs by dividing overlapping periods by the number of drugs and summing the times that a particular drug was used. Section 4 describes how this approach can be used to generalize the one-drug-at-a-time model to a multiple drug scenario. In effect, this gives an estimate of the time to evolve resistance for each drug in the absence of any other drug.

Approach 2: Time to evolve resistance from earliest drug arrival to first drug failure

Alternatively, we can consider time to evolve resistance as the time from the earliest drug arrival to the time that resistance is first observed to any drug in an overlapping period. Using this approach, estimates of time to resistance are not necessarily for a specific drug, but instead reflect the time to resistance for a group of drugs (and the factors experienced by these drugs) that are in use during an overlapping period.

Simulating the current and future state of antimalarial supply

Antimalarial drug supply was simulated using the parameter estimates from the antimalarial data as described above. Figure S2 shows how periods of time to failure and drug availability observed from 1930-2012 in the antimalarial data agree with a characteristic simulation of the current state of drug supply. We can also envision what might happen to antimalarial supply in the future if we slowed resistance or enhanced drug development. In this case, we chose to compare an increase of 2 years in the mean time to evolve resistance with a decrease of 2 years in the mean time between drug arrivals. Figure S3 shows that increasing the mean time to evolve resistance results in significantly longer periods of time to failure, which also means that drugs will be available for a longer fraction of time over the next 200 years.

Again we stress, however, that these simulation results are merely meant to be an illustrative example. The findings from the model are independent of the details of this simulation, and apply for any distribution of time to resistance, regardless of its shape or parameter values. The model also allows for the use of any (consistent) measure of resistance or drug failure since time to resistance is treated as a random process. In this example, the data report the first observation of resistance but we might wish to measure drug lifespan as the time until resistance is observed at some threshold frequency instead.

2 – Analysis

All models presented in the text are analogous to models from queueing theory. There are abundant results available for analyzing their behavior [52]. In what follows we make use of results found in [52].

Exponentially Distributed Time to Evolve Resistance

When the time to evolve resistance is exponentially distributed, the drug supply model is analogous to an M/M/1 queue [52]. Using $f_T(t)$ to denote the probability density of the time to failure, and $\tilde{f}_T(z)$ for its *Laplace-Stieltjes transform* (LST), standard results [52] demonstrate that

$$\tilde{f}_T(z) = \frac{\alpha + \beta + z - \sqrt{(\alpha + \beta + z)^2 - 4\beta\alpha}}{2\alpha} \quad \text{(Equation 1)}$$

where $E[D]=1/\alpha$ and $E[L]=1/\beta$ (Equation 1 is also derived below in the case of an arbitrary distribution for the time to evolve resistance).
Inverting the transform gives the density [52]

$$f_T(t)=\sqrt{\frac{\beta}{\alpha}}\,\frac{e^{-(\alpha+\beta)t}I_1(2\sqrt{\alpha\beta}t)}{t} \qquad \text{(Equation 2)}$$

where $I_1(x)$ is a modified Bessel function of the first kind with order 1. Specifically, $I_1(x)$ can be defined by the integral formula

$$I_1(x)=\frac{1}{\pi}\int_0^{\pi}e^{x\cos\theta}\cos\theta\,d\theta. \qquad \text{(Equation 3)}$$

Drug availability, ρ, is the long run proportion of time an effective drug is available. In the context of queueing theory this is referred to as the "load" or "traffic intensity". Standard results [52] show that

$$\rho=E[L]/E[D]. \qquad \text{(Equation 4)}$$

Equation 4 can be understood by recognizing that drug availability is the long-term time spent with at least one effective drug (which is, on average, $E[T]$) divided by the length of the cycle (which is, on average, $E[T]+E[D]$). In other words, the ratio

$$\frac{E[T]}{E[T]+E[D]}.$$

Using Equation 2 we can calculate $E[T]=E[L]/(1-E[L]/E[D])$. Substituting this into the above ratio then gives Equation 4.

We can now determine the effect of each intervention on Equation 2 and Equation 4. The density (Equation 2) is a decreasing function of *t* and changes in $E[D]=1/\alpha$ or $E[L]=1/\beta$ affect the density differently. For example,

$$\lim_{t\to 0}f_T(t)=\frac{1}{E[L]},$$

shows that increasing the expected time to evolve resistance (either additively or multiplicatively) decrease the probability density at $t=0$ whereas decreasing the expected time between drug arrivals has no effect at this point. Consequently, given that the density function is continuous, interventions that target evolution will shift more probability density from near zero to larger values of *T* than will interventions that target drug development.

For the drug availability, Equation 4, we consider additive and multiplicative changes in turn. An additive change of size *x* will either add *x* to the mean time to evolve resistance

or subtract x from the mean time between drug arrivals. A multiplicative change of size $y>1$ will either multiply the mean time to evolve resistance by y or divide the mean time between drug arrivals by y.

For additive changes of size x the inequality

$$\frac{E[L]+x}{E[D]} > \frac{E[L]}{E[D]-x}$$

holds for all x that satisfy the assumption that ρ is less than 1. Alternatively, multiplicative changes result in equal benefits to drug availability as

$$\frac{E[L]\times y}{E[D]} = \frac{E[L]}{E[D]/y}.$$

Therefore, when evolution of drug resistance is slowed, effective treatment will be available for a fraction of time greater than or equal to the effect from enhancing drug development.

Arbitrary Distribution for the Time to Evolve Resistance

When time to evolve resistance has an arbitrary distribution, the drug supply model is analogous to an M/G/1 queue [52]. We can derive an equation for the *Laplace-Stieltjes transform* (LST) of the time to failure distribution as follows.

Any time to failure period begins with the arrival of a new drug into the portfolio. While this drug is in use, additional new drugs might be added to the portfolio. Suppose, for example, there are K additional drugs added during the time that the first drug is being used (K is a random variable). Each of these additional drugs will eventually be used, and each will, themselves, spawn a time to failure period that has the same distribution as that of the first drug. In this way we can write

$$T = L + T_1 + \cdots + T_K$$

where L is the time to evolve resistance for the first drug, and the T_i are the time to failure periods for the additional K drugs that arrive before the first drug is abandoned. Using $f_L(t)$ and $f_T(t)$ to denote the probability densities for the time to evolve resistance and time to failure respectively, the LST of $f_T(t)$ is $\tilde{f}_T(z) = E[e^{-zT}]$. Conditioning on the lifespan of the first drug we obtain

$$\tilde{f}_T(z) = \int_0^\infty f_L(t) E[e^{-zT} \mid L = t]dt,$$

and further conditioning on the number of new drug arrivals, K, during this time gives

$$\begin{aligned}\tilde{f}_T(z) &= \int_0^\infty f_L(t)\left(\sum_{k=0}^\infty E[e^{-z(L+T_1+\cdots+T_K)} \mid L=t, K=k]P(K=k \mid L=t)\right)dt \\ &= \int_0^\infty f_L(t)e^{-zt}\left(\sum_{k=0}^\infty E[e^{-z(T_1+\cdots+T_k)}]P(K=k \mid L=t)\right)dt \\ &= \int_0^\infty f_L(t)e^{-zt}\left(\sum_{k=0}^\infty E[e^{-z(T_1+\cdots+T_k)}]\frac{(\alpha t)^k e^{-\alpha t}}{k!}\right)dt\end{aligned}$$

where the final equality makes use of the fact that *K* is Poisson distributed. Now, using the fact that the T_i are independent and identically distributed, we obtain

$$\begin{aligned}\tilde{f}_T(z) &= \int_0^\infty f_L(t)e^{-zt}\left(\sum_{k=0}^\infty E[e^{-zT}]^k \frac{(\alpha t)^k e^{-\alpha t}}{k!}\right)dt \\ &= \int_0^\infty f_L(t)e^{-zt}\left(\sum_{k=0}^\infty \tilde{f}_T(z)^k \frac{(\alpha t)^k e^{-\alpha t}}{k!}\right)dt.\end{aligned}$$

Finally, noting that $\sum_{k=0}^\infty z^k \frac{(\alpha t)^k e^{-\alpha t}}{k!} = e^{(z-1)\alpha t}$, this last expression simplifies to

$$\begin{aligned}\tilde{f}_T(z) &= \int_0^\infty f_L(t)e^{-zt}e^{(\tilde{f}_T(z)-1)\alpha t}dt \\ &= \int_0^\infty f_L(t)e^{-(z+\alpha-\alpha\tilde{f}_T(z))t}dt\end{aligned}$$

or

$$\tilde{f}_T(z) = \tilde{f}_L\left(z+\alpha-\alpha\tilde{f}_T(z)\right) \qquad \text{(Equation 5)}$$

Equation 5 is Takács functional equation relating the LST of the distribution of time to failure, *T*, to the LST of the distribution of time to evolve resistance, *L*.

We can now calculate any moment of interest for the distribution of the time to failure, in terms of the moments of the distribution of time to evolve resistance. In particular, $E[T] = -\tilde{f}_T'(0)$, and therefore

$$E[T] = \frac{E[L]}{1 - E[L]/E[D]}. \qquad \text{(Equation 6)}$$

Notice that this is identical to the expression for *E*[*T*] obtained in the case of exponentially distributed time to evolve resistance, and therefore Equation 4 for drug availability remains valid even when the time to evolve resistance has an arbitrary distribution.

Incidentally, we can also derive Equation 1 from Equation 5 by using the fact that, when L is exponentially distributed with parameter β, the LST of L is $\tilde{f}_L(z) = \beta / (\beta + z)$. Substituting this into Equation 5 gives

$$\tilde{f}_T(z) = \frac{\beta}{\beta + z + \alpha - \alpha \tilde{f}_T(z)}.$$

Solving this quadratic equation for $\tilde{f}_T(z)$ gives Equation 1.

When the distribution of L is arbitrary, we can consider the effect that additive and multiplicative changes have on any moment of the time to failure distribution. For the purposes of this analysis, we have chosen to focus on the first moment, the expected time to failure, $E[T]$ (Equation 6). The expected time to failure can be broken up into two parts: the average number of drugs used before failure, $E[N] = (1 - E[L]/E[D])^{-1}$ and the average lifespan of each drug, $E[L]$, such that $E[T] = E[N] \times E[L]$. The likelihood that another drug arrives before failure will increase as a result of slowing the time to evolve resistance or decreasing the time between drug arrivals. An additive change of size x increases the average number of drugs used before failure and, in particular, the inequality

$$\left(1 - \frac{E[L] + x}{E[D]}\right)^{-1} > \left(1 - \frac{E[L]}{E[D] - x}\right)^{-1}$$

holds for all x that satisfy the assumption that ρ is less than 1. Alternatively, there is an equivalent increase in the average number of drugs used before failure when evolution is slowed and drug development is enhanced multiplicatively since

$$\left(1 - \frac{E[L] \times y}{E[D]}\right)^{-1} = \left(1 - \frac{E[L]}{E[D]/y}\right)^{-1}.$$

This shows that increasing the mean time to evolve resistance will result in at least as many drugs used before failure as decreasing the mean time between drug arrivals. In addition, since increasing the mean time to evolve resistance also increases average drug lifespan, the total effect on average time to failure is that much greater when resistance is slowed than when drug development is enhanced.

Second Moment of Time to Failure

Our analysis in the main text has focused on the expected value of time to failure E[T] as a measure of system performance. Higher moments may be used to investigate additional properties of the distribution of time to failure. For instance, studying the second moment of time to failure is useful in describing the variability in the length of periods for which treatment is available.

Using Takács functional equation (Equation 5) and $E[T^2] = \tilde{f}_T''(0)$ gives

$$\tilde{f}_T''(0) = \tilde{f}_L''(0)(1 - \alpha\tilde{f}_T'(0))^2 - \alpha\tilde{f}_L'(0)\tilde{f}_T''(0).$$

Solving this equation for $\tilde{f}_T''(0)$ and noting that $\sigma_X^2 = E[X^2] - E[X]^2$ we obtain

$$\sigma_T^2 = \frac{\sigma_L^2 + E[L]^2 \rho}{(1-\rho)^3}.$$

We have shown elsewhere that the increase in ρ is equivalent for multiplicative changes in E[D] and E[L] and greater for additive changes in E[L]. Therefore, when all else is equal, there is a greater increase in the variance of T when E[L] is increased compared to when E[D] is decreased. Thus, slowing evolution of resistance increases both the expected time to failure as well as its variability compared to enhancing drug development.

3 – Numerical Simulations

Gamma Distributed Drug Inter-arrival Time and Drug Lifespan

We consider a scenario when time between drug arrivals and time to evolve resistance are not exponentially distributed. We used a gamma distributed drug inter-arrival time, D, and gamma distributed drug lifespan, L. The gamma distribution is parameterized by a shape parameter, k, and a rate parameter, which we denote by α and β for the drug inter-arrival time and drug lifespan respectively. The expected time between drug arrivals is therefore $E[D] = k\alpha$. Similarly, the expected time to evolve resistance is $E[L] = k\beta$. We numerically calculated time to failure and drug availability for an additive change and a multiplicative change that increased E[L] or that decreased E[D], and compared the results to baseline conditions.

Numerical results show the effect on average time to failure and drug availability as a result of these two manipulations (Table S2). In both cases, average time to failure increases; however, we observe a greater increase when E[L] is extended than when E[D] is decreased. For an additive change, there was also a greater increase in drug availability when E[L] increased than when E[D] decreased, while for a multiplicative change, drug availability was increased by the same amount, which agrees with the analytical results (Table S2).

Variable Rate of Drug Arrival

We will also consider a variable rate of drug arrival so that drugs arrive at a normal rate, α, when there are lots of drugs in the drug portfolio and a fast rate, θ, when there are 2 or fewer effective drugs available at the time of the last drug arrival. The normal rate of drug arrival was varied to determine the effect of speeding up drug development. As

before, these results were compared against the effect of slowing evolution of resistance. In these simulations, the proportion of time that drugs arrived at their respective rates was also considered.

The available empirical evidence suggests that annual drug production of 'new molecular entities' has been constant for over 55 years, remaining unchanged in spite of increased investment [31]. Even so, by including a fast rate, the time between drug arrivals is not necessarily longer than the time it takes resistance to evolve, relaxing the $E[D]>E[L]$ assumption of the previous model.

Numerical results show that slowing the evolution of resistance benefits average time to failure and drug availability more than speeding up the normal rate of drug arrival (Table S3). Slowing evolution of drug resistance also decreased the proportion of time that drugs arrived according to a fast rate (Table S3). This suggests that drug arrivals were occurring close to a maximum rate, so that any further increase in the normal rate of drug arrival has less of an effect on system performance. In contrast, when evolution was slowed, a normal rate of drug arrival was used for a greater proportion of time implying that the average rate of drug arrival slowed down when $E[L]$ increased.

4 – Generalizing to Multiple Drugs

Thus far, we have presented a model of drug supply that assumes one drug or drug therapy is used at a time. While this assumption is supported by current treatment guidelines and practice for malaria (wherein a single first line therapy is recommended for treatment and replaced when resistance emerges [21,25]) in general, it is likely that more than one drug will be used to treat a disease when available. Note, by this we mean that individuals are still given a single drug, but different individuals might be given different drugs simultaneously.

To begin it is helpful to first consider how simultaneous drug use affects the total time required to evolve drug resistance for a set of drugs, relative to that required if the drugs in this set were used one at a time (i.e., sequentially). There are three possibilities: (i) conservation of evolution, (ii) compression of evolution, and (iii) expansion of evolution. These are defined as follows.

Suppose there are n drugs currently available. We say that there is conservation of evolution if the total time it takes for resistance to evolve to all n drugs when used simultaneously is the same as the time required for resistance to evolve to all n drugs when used sequentially. Alternatively, we say that there is compression of evolution if the time taken under simultaneous drug use is shorter, and expansion of evolution if it is longer. We will show that this provides a heuristic framework with which to examine multiple drug use.

It can be shown, using results from work-conserving queueing theory [53], that a model with simultaneous drug use is formally equivalent to a one-drug-at-a-time model when

there is conservation of evolution. We propose a mechanistic basis by which this occurs. Suppose that the rate of evolution for individual drugs depends on the number of drugs in use, n, such that the rate of evolution for each drug is slowed by a factor $1/n$ relative to the rate of resistance evolution if it were the only drug in use. Then it can be shown that the distribution of time to failure is unchanged from a one drug at a time model, as this is analogous to a work-conserving queue [53]. In a similar way, we can account for drugs used at different frequencies by assuming that the rate of evolution of resistance to each is slowed by a factor equal to its frequency of use.

These ideas reveal that, under certain conditions (namely, conservation of evolution) the results of the simple model of the text are identical to those for a model that allows simultaneous multiple drug use. Therefore, increasing the mean time to evolve resistance is more effective than decreasing the mean time between drug arrivals in an evolution-conserving scenario of multiple drug use. This also provides a natural point of departure for exploring cases in which evolution is not conservative.

When multiple drug use results in compression of evolution, this means that there are interactions among drugs that result in a reduction of the time required to evolve resistance. This effect might arise from cross-resistance, wherein the mechanism of resistance to one drug also confers resistance to another drug, effectively reducing its lifespan. This means that it is more important to slow resistance than to enhance drug development, as simply using more drugs will reduce the lifespan of each drug.

Alternatively, when multiple drug use results in the expansion of evolution, this means that there are interactions among drugs that bring about an increase in the time required for resistance evolution. At the population level, drug mixing has been suggested as a way of spatially varying the drug environment so that it is more difficult for microbes to evolve resistance to any one drug [54].

5 – Supplementary Tables

Table S1. Data for antimalarials and antibiotics. The values indicate the time of drug introduction and first observation of drug resistance. The reference for each drug is provided; all data is from published sources.

Drug	Drug Type	Introduced	Resistance	Source
Chloroquine	Antimalarial	1933.5	1957	(a)
Sulfadoxine-pyrimethamine	Antimalarial	1940	1953	(a)
Proguanil	Antimalarial	1948	1949	(b)
Pyrimethamine	Antimalarial	1951.5	1952.5	(c)
Mefloquine	Antimalarial	1977	1982	(d)
Halofantrine	Antimalarial	1988	1992	(b)
Atovaquone	Antimalarial	1996	1996	(d)
Atovaquone-proguanil	Antimalarial	1996.5	2001.5	(c)
Artemisinin	Antimalarial	2000	2009	(a)
Sulfonamides	Antibiotic	1930	1940.5	(e)
Penicillin	Antibiotic	1943	1946	(f)
Streptomycin	Antibiotic	1943	1959	(f)
Chloramphenicol	Antibiotic	1947	1959	(f)
Tetracycline	Antibiotic	1948	1953	(f)
Erythromycin	Antibiotic	1953	1968	(g)
Methicillin	Antibiotic	1960	1961	(f)
Cephalosporins	Antibiotic	1960.5	1969.5	(e)
Ampicillin	Antibiotic	1961	1973	(f)
Gentamicin	Antibiotic	1967	1970	(h)
Vancomycin	Antibiotic	1972	1988	(g)
Oxyimino-beta-lactams	Antibiotic	1981	1983	(h)
Ceftazidime	Antibiotic	1985	1987	(g)
Imipenem	Antibiotic	1985	1998	(g)
Levofloxacin	Antibiotic	1996	1996	(g)
Linezolid	Antibiotic	1999.5	2003.5	(e)
Daptomycin	Antibiotic	2003.5	2005	(e)
Ceftaroline	Antibiotic	2010	2011	(g)

(a) Shetty, P. 2012 Malaria – the numbers game. *Nature* **484**, S14–S15.
(b) Thayer, A. M. 2005 Fighting malaria. *Chemical and Engineering News* **83**, 69–82.
(c) Hyde, J.E. 2005 Drug-resistant malaria. *Trends Parasit.* **21**, 494–498.
(d) Wongsrichanalai, C., Pickard, A. L., Wernsdorfer, W. H. & Meshnick, S. R. 2002 Epidemiology of drug-resistant malaria. *Lancet Infect. Dis.* **2**, 209–218.
(e) Clatworthy, A. E., Pierson, E. & Hung, D. T. 2007 Targeting virulence: a new paradigm for antimicrobial therapy. *Nat. Chem. Biol.* **3**, 541–548.
(f) Palumbi, S. R. 2001 Humans as the world's greatest evolutionary force. *Science* **293**, 1786–1790.
(g) CDC 2013 Antibiotic resistant threats in the United States, 2013. Georgia: U.S. Department of Health and Human Services Centers for Disease Control and Prevention.
(h) Jacoby, G. A. 2009 History of drug-resistant microbes. In *Antimicrobial drug resistance* (ed. D. L. Mayers), pp. 3–7. New York: Humana Press.

Table S2. Results from replicate simulations (n = 100) with gamma distributed drug inter-arrival time and drug lifespan over a time interval of 20000 years. Performance measures are given as averages over all replicates. Baseline conditions are given as $k = 2$, $\alpha = 1/5$, $\beta = 1/2$. An additive change affects the mean time to evolve resistance or mean time between drug arrivals by 2 years, while a multiplicative change doubles the mean time to resistance or halves the mean time between drug arrivals.

	Baseline conditions	*Additive change*		*Multiplicative change*	
(Mean time between drug arrivals, Mean time to evolve resistance)	10, 4	10, 6	8, 4	10, 8	5, 4
**Average drug availability**	0.40	0.60	0.50	0.80	0.80
**Average time to failure (years)**	5.35	11.40	6.30	28.88	14.58
**Average down time (years)**	8.05	7.57	6.24	7.28	3.64

Table S3. Results from replicate simulations (n = 100) with a variable rate of drug arrivals over a time interval of 20000 years. Performance measures are given as averages over all replicates. The mean time between drug arrivals was 3 years ($\theta = 1/3$) when there were 2 or fewer drugs in the portfolio at the time of the last drug arrival. Baseline conditions are given as $\alpha = 1/10$, $\beta = 1/4$. An additive change increases the mean time to evolve resistance by 2 years or decreases the mean time between drug arrivals under a normal rate by 2 years, while a multiplicative change doubles the mean time to resistance or halves the mean time between drug arrivals under a normal rate.

(Mean time between drug arrivals, Mean time to evolve resistance)	*Baseline conditions*	*Additive change*		*Multiplicative change*	
	10, 4	10, 6	8, 4	10, 8	5, 4
**Average drug availability**	0.69	0.84	0.75	0.93	0.90
**Average time to failure (years)**	12.96	33.69	14.97	100.09	31.64
**Average down time (years)**	5.77	6.66	5.94	7.35	3.71
**Average proportion of time fast rate used**	0.31	0.17	0.30	0.07	0.18

6 – Supplementary Figures

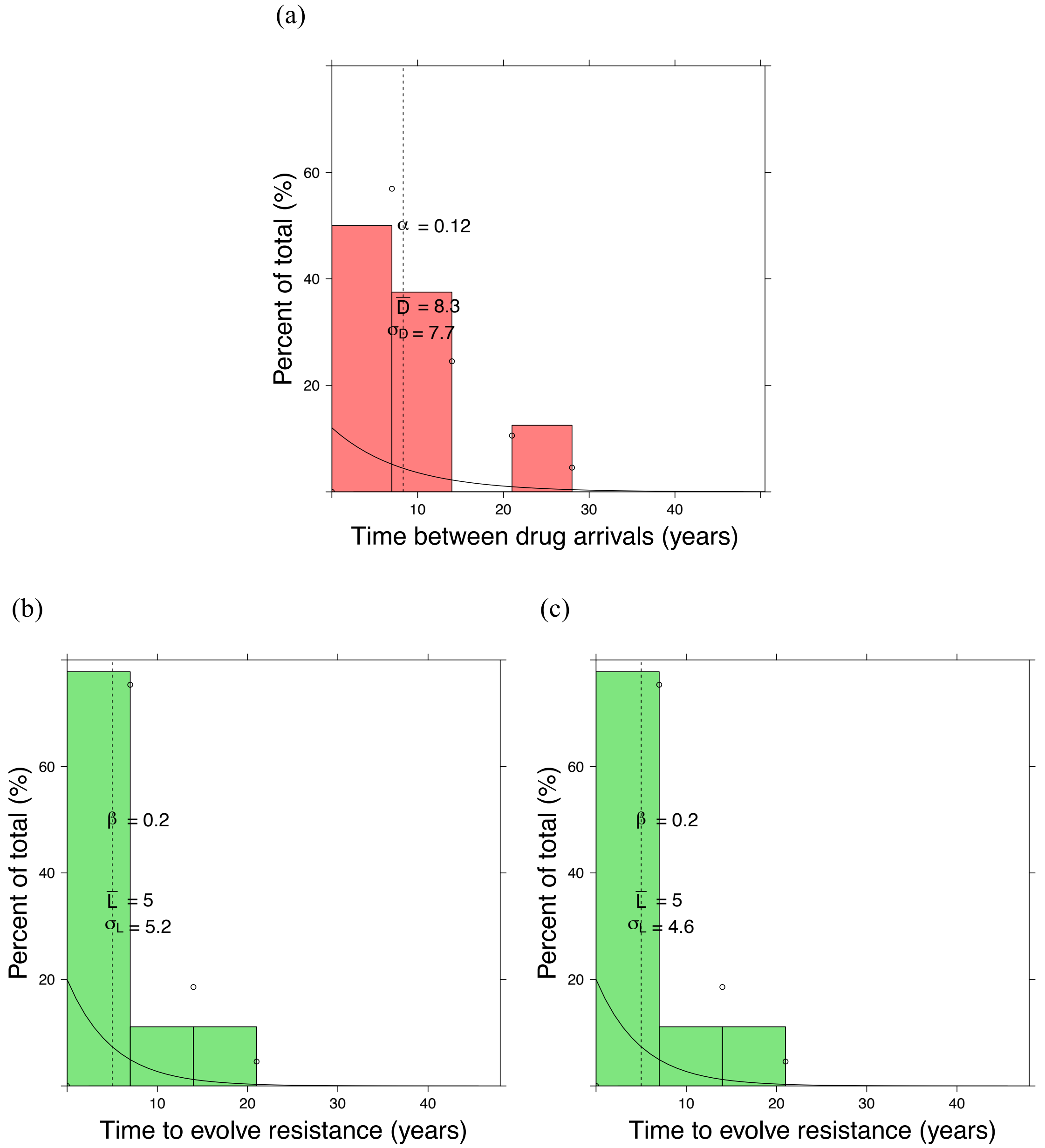

Figure S1. Histograms of drug supply parameters from antimalarial data. (a) The distribution of time between drug arrivals (α = 0.12, $\bar{D}$ = 8.3 years, σ_D = 7.7 years). The distribution of time to evolve resistance was estimated via two approaches: (b) by dividing overlapping regions by the number of drugs (β = 0.2, $\bar{L}$ = 5 years, σ_L = 5.2 years) or (c) using the time from earliest drug arrival to first drug failure (β = 0.2, $\bar{L}$ = 5 years, σ_L = 4.6 years). Using the estimate of the rate parameter in each panel, the solid line is the exponential density plot and the open circles give the probability (%) within the interval defined by the width of each bar. The dashed line is the average value of the variable of interest.

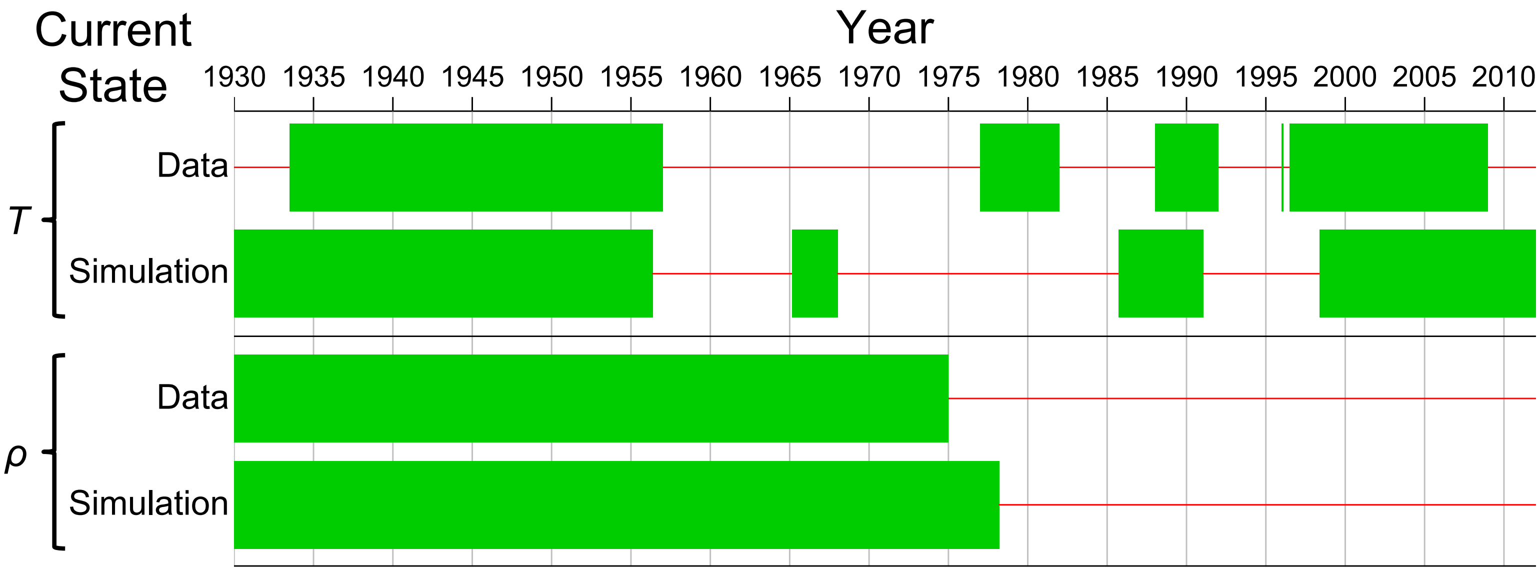

Figure S2. The current state of antimalarial supply. This shows the time to failure (T; green bars) and drug availability (ρ) observed from data and from simulation using parameter estimates ($E[L] = 5$, $E[D] = 8.3$).

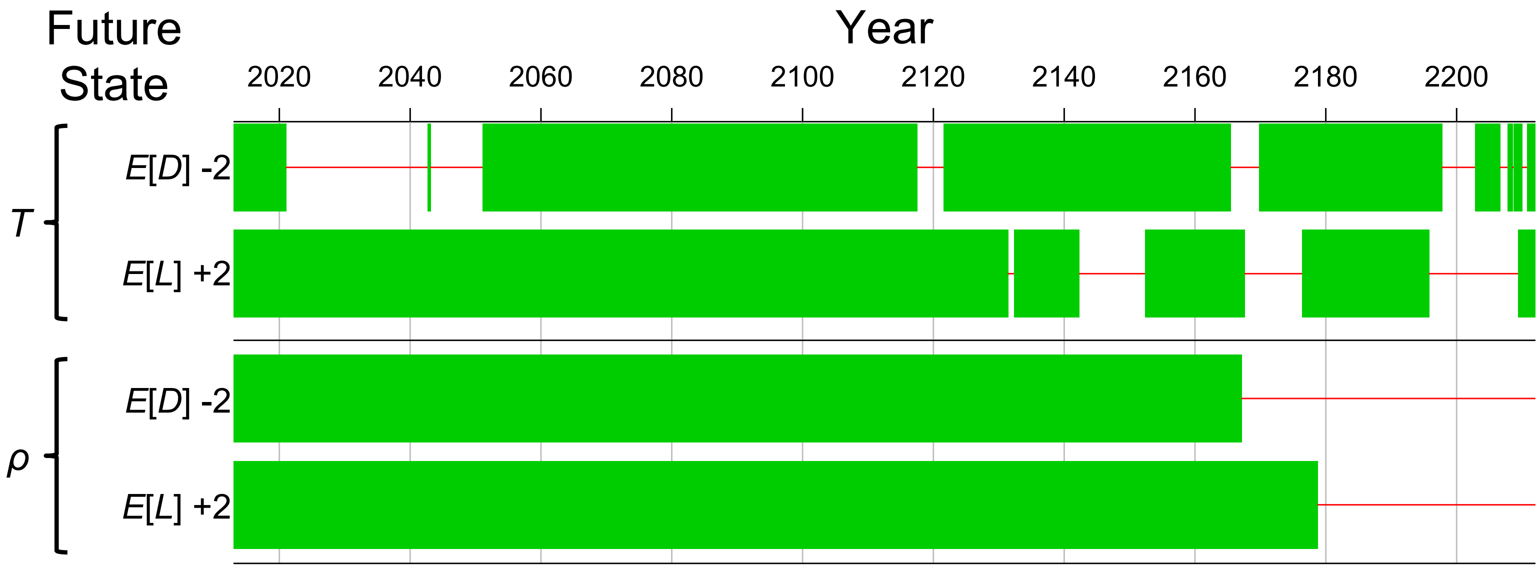

Figure S3. Characteristic realizations of future antimalarial supply. A simulation over the next 200 years when the mean time between drug arrivals is reduced by 2 years or the mean time to evolve resistance is increased by 2 years (compared to initial conditions: $E[L] = 5$, $E[D] = 8.3$).
